# Establishment and characterization of persistent *Pseudomonas aeruginosa* infections in air–liquid interface cultures of human airway epithelial cells

**DOI:** 10.1128/iai.00603-24

**Published:** 2025-02-18

**Authors:** Safaa Bouheraoua, Sven Cleeves, Matthias Preusse, Mathias Müsken, Peter Braubach, Maximilian Fuchs, Christine Falk, Katherina Sewald, Susanne Häussler

**Affiliations:** 1 Institute for Molecular Bacteriology, TWINCORE, Centre for Experimental and Clinical Infection Research, Hannover, Germany; 2 Fraunhofer Institute for Toxicology and Experimental Medicine, Hannover, Germany; 3 Department of Molecular Bacteriology, Helmholtz Center for Infection Research 28336 , Braunschweig, Germany; 4 Central Facility for Microscopy, Helmholtz Center for Infection Research, Braunschweig, Germany; 5 Institute for Pathology, Hannover Medical School, Hannover, Germany; 6 Institute for Transplantation Immunology, Hannover Medical School, Hannover, Germany; 7 Biomedical Research in Endstage and Obstructive Lung Disease Hannover (BREATH), Hannover, Germany; 8 Fraunhofer Cluster of Excellence Immune-Mediated Diseases CIMD, Hannover, Germany; 9 Department of Clinical Microbiology, Copenhagen University Hospital-Rigshospitalet 683301 , Copenhagen, Denmark; 10 Cluster of Excellence RESIST (EXC 2155), Hannover Medical School, Hannover, Germany; University of California Davis, Davis, California, USA

**Keywords:** *Pseudomonas aeruginosa*, airway infection model, bacterial infection, host–pathogen interactions, 3D *in vitro* models, persistent infections, lung infection

## Abstract

Bacteria exhibit distinct behaviors in laboratory settings compared to infection environments. The presence of host cells induces changes in bacterial activity, while pathogens trigger immune responses that shape the microenvironment. Studying infection dynamics by microscopy, cytokine screening, and dual RNA sequencing in an air–liquid interface model, we found that prolonged *Pseudomonas aeruginosa* colonization of airway epithelium led to a pro-inflammatory response, consistent across *P. aeruginosa* strains, despite differences in the dynamics of this response. Concurrently, *P. aeruginosa* formed non-attached aggregates on the apical side of the cell layer and upregulated genes involved in biofilm formation and virulence. Notably, there was remarkable resemblance between the *P. aeruginosa* transcriptional profile in our model and that previously reported upon host cell contact. Developing a platform that replicates host microenvironments is vital not only for gaining deeper insights into the interplay between host and pathogen but also for evaluating therapeutic strategies in conditions that closely mirror clinical environments.

## INTRODUCTION

The ubiquitous gram-negative bacterium *Pseudomonas aeruginosa* is categorized as an ESKAPE pathogen (*Enterococcus faecium*, *Staphylococcus aureus*, *Klebsiella pneumoniae*, *Acinetobacter baumannii*, *Pseudomonas aeruginosa*, and *Enterobacter* spp,) belonging to a subset of highly virulent, often multidrug-resistant pathogens known for their role in nosocomial or hospital-acquired infections (1). As an opportunistic pathogen, *P. aeruginosa* can cause severe acute life-threatening infections throughout different human body sites. It is furthermore involved in the establishment of chronic infections, associated with the formation of biofilms. Growth within biofilm structures protects the bacteria from the human immune system and antimicrobial therapy. Once biofilms are formed, it becomes nearly impossible to eradicate the infection (2). Chronic wounds and the respiratory tract of patients with impaired lung function, especially those suffering from cystic fibrosis (CF), bronchiectasis, and chronic obstructive pulmonary disease (2), are particularly vulnerable to chronic *P. aeruginosa* infections. These infections can persist for years, leading to extensive lung tissue damage, and are often associated with poor patient outcomes (3). Moreover, lower respiratory tract infections emerged as the leading cause of deaths attributed to antimicrobial resistance worldwide, with *P. aeruginosa* ranking among the top six contributing pathogens (4).

*P. aeruginosa* virulence has been well analyzed using numerous *in vitro* and *in vivo* models of acute infections (5–7). Complex *in vitro* cell-based models such as air–liquid interface (ALI) epithelial cell cultures have been used to study the role of efflux pumps (8), virulence factors (9), viral co-infections (10–12), and bacteriophage treatments (13) in *P. aeruginosa* infections. However, there are fewer models available to study chronic infections (14–17) and there remains a deficiency in *in vitro* models capable of mimicking *P. aeruginosa* colonization in the human airways while enabling the study of persistent infections over time. Models of persistent infections need to enable sufficient host–pathogen interaction to study the development of subsequent time-resolved responses. Unfortunately, most infection studies using two-dimensional or three-dimensional (3D) cell culture models are limited to just a few hours (8, 9, 12, 18–25) due to the pronounced cytotoxicity exhibited by well-characterized reference strains like PAO1 and PA14 (26, 27)

In this study, we aimed to enhance our understanding of how host-bacteria interactions evolve over time during persistent bacterial contact with the human airway epithelium. To achieve this, we established an accessible and reproducible human cell-based airway infection model that emulates key factors of the host–bacteria interface, including sustained contact between host and bacteria to enable the elicitation of robust immune responses and the development of bacterial adaptive strategies. We developed our model at the ALI, which allows for the formation of a 3D polarized epithelium, enabling differential manipulation of the apical and basolateral compartments. We selected Calu-3 cells, a human lung epithelial cell line commonly used in research and drug development (28, 29) as well as in *P. aeruginosa* infection studies at the ALI (10, 13, 30) alongside well-described lab strains of *P. aeruginosa*. To prevent bacterial overgrowth and cytotoxicity, we applied tobramycin, a commonly used antibiotic (31–33) to maintain the model, thereby reflecting a common feature of the microenvironment in chronic *P. aeruginosa* infections. Additionally, the model was validated using primary normal human bronchial epithelial (NHBE) cells at the ALI as well as CF strains of *P. aeruginosa*.

The successful maintenance of an undisturbed host–pathogen interface allowed us to study infection dynamics over time by microscopy, cytokine screening, and dual RNA sequencing. Our findings illustrate the complex scenario of prolonged airway colonization, emphasizing the remarkable adaptability of *P. aeruginosa* to challenging infection environments. Generating a platform that replicates the host microenvironment during prolonged *P. aeruginosa* contact is not only crucial for gaining deeper insights into the interplay between host and pathogen factors influencing disease progression but also offers the opportunity to evaluate innovative therapeutic approaches in settings that closely mimic clinical conditions.

## RESULTS

### Prolonged infection of respiratory epithelial cells with *P. aeruginosa*

**Fig 1:**
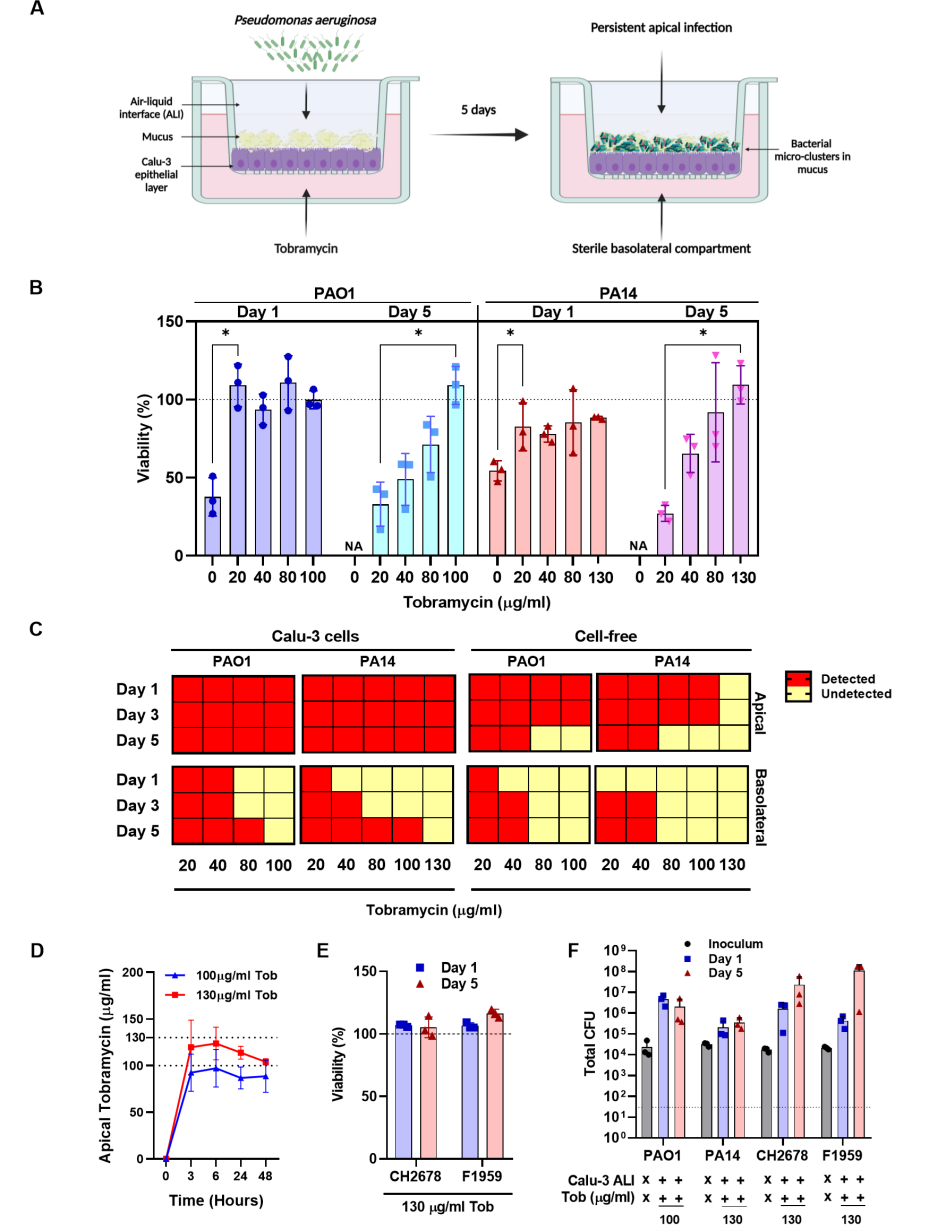
Prolonged infection of Calu-3 cells with *P. aeruginosa* in the presence of tobramycin. (**A**) Schematic diagram of Calu-3 infection with *P. aeruginosa* at ALI (created with BioRender.com). (**B**) Viability of *P. aeruginosa* PAO1- and PA14-infected and tobramycin-protected Calu-3 cells on day 1 and day 5 p.i. compared to uninfected controls as measured by Calcein AM viability staining. Significance was determined via *t*-test, * indicates *P*-value <0.05, NA indicates data not available. (**C**) Presence of bacteria in the apical and basolateral side of Calu-3 cells at increasing tobramycin concentrations (left panel). The same experimental set-up was used without the presence of Calu-3 cells (right panel). Red squares indicate detected bacteria and yellow squares indicate no detected bacteria after plating of 3 µL on lysogeny broth (LB) agar plates. (**D**) Estimated concentration of tobramycin in the apical layer of Calu-3 epithelial layer basolaterally treated with either 100 µg/mL or 130 µg/mL tobramycin as determined by spot assay (see Materials and Methods for details). (**E**) Viability of Calu-3 cells infected with *P. aeruginosa* CF strains CH2678 and F1959 and protected with tobramycin concentrations of 130 µg/mL compared to uninfected controls as measured by Calcein AM viability staining. (**F**) Total apical colony-forming units (CFUs) of the different *P. aeruginosa* strains from the inoculum and on day 1 and day 5 after Calu-3 cell infection. Calu-3 cells were protected by tobramycin concentrations of either 100 µg/mL or 130 µg/mL. Dotted line indicates a lower limit of detection. All data were obtained from three biological replicates per condition.

The successful infection of Calu-3 epithelial cells with *P. aeruginosa* at ALI requires protecting the epithelial cells from cell death caused by overwhelming infection , while maintaining apical bacterial presence. We achieved this protection by adding a carefully optimized concentration of tobramycin to the basolateral compartment, after infecting the Calu-3 cells apically with *P. aeruginosa* (Fig. 1A). The optimal concentration of tobramycin needed to maintain an infection for 5 days varied depending on the *P. aeruginosa* strain and was determined through a titration experiment. This experiment identified the tobramycin concentration required to sustain host cell viability throughout a 5-day infection period (Fig. 1B), while also limiting the breakthrough of bacteria from the apical side of the cells into the basolateral medium (Fig. 1C). Tobramycin decreased host cell death and bacterial breakthrough in a dose-dependent manner, with higher concentrations of tobramycin needed at day 5 post infection (p.i.) compared to day 1 (Fig. 1B and C). The optimal tobramycin treatment was defined to be 100 µg/mL for PAO1 and 130 µg/mL for PA14. This concentration is about 50× the minimum inhibitory concentration (MIC) of both the strains of 2 µg/mL (Fig. S1A). At these concentrations, bacteria were detected at the apical side but not in the basolateral compartment, indicating the integrity of the epithelial membrane (Fig. 1C).

Interestingly, in the absence of Calu-3 cells, lower concentrations of tobramycin were sufficient to prevent bacterial presence in the apical and basolateral sides. This suggests an enhanced bacterial recalcitrance to tobramycin treatment in the presence of host cells (Fig. 1C). This observation is in line with previous studies where *P. aeruginosa* grown on epithelial cells became more resistant to killing by aminoglycosides (19, 24, 34). Furthermore, at these optimized concentrations, tobramycin diffused through the Calu-3 epithelial layer into the apical side of the cells by 3 hours post treatment and remained relatively stable for 48 hours (Fig. 1D). Accordingly, exchange of the tobramycin-containing basolateral medium every 48 hours efficiently protected the epithelial cells from cell death by the overwhelming infection of the two reference strains PA14 and PAO1 (Fig. 1B), while maintaining apical bacterial presence over extended periods (Fig. 1F).

Comparable results were found in primary NHBE cells infected with PAO1 and treated with tobramycin at the basolateral side. Here, with the more complex nature of the pseudostratified epithelium, a lower concentration of 20 µg/mL tobramycin was sufficient to maintain host cell viability for up to 3 days p.i. (Fig. S2A) and preserve the presence of bacteria (Fig. S2B), thus highlighting the importance of adequate titration.

Having demonstrated that ALI cultures of Calu-3 as well as NHBE cells can be successfully infected with *P. aeruginosa* reference strains for prolonged time periods, we wanted to confirm that our experimental cell culture set-up can also be used with other strains of *P. aeruginosa*. We therefore tested two clinical *P. aeruginosa* isolates, CH2678 and F1959, from our collection (35). Both strains were recovered from CF lungs, are PAO1-like (36, 37), are non-motile (37, 38), and exhibited low virulence in the *Galleria mellonella* infection model (39). CH2678 is a *lasR* mutant while F1959 has a functional *lasR*, a quorum sensing receptor protein that activates the transcription of a large regulon including many virulence genes (40). Interestingly, the same tobramycin concentration of 130 µg/mL was needed to maintain basolateral sterility in infections with both clinical isolates as the PA14 strain. This could be due to the higher MIC to tobramycin in the two clinical isolates of 4 µg/mL for CH2804 and 32 µg/mL for F1959, as compared to PAO1 and PA14 (2 µg/mL) (Fig. S1A). Using the optimized set-up, we observed maintained host cell viability (Fig. 1E) and increasing apical bacterial load following the basolateral addition of tobramycin at a concentration of 130 µg/mL (Fig. 1F), indicating that our protocol can be applied to other *P. aeruginosa* strains. Strain-adapted tobramycin concentrations were used for all further infection experiments of Calu-3 cells.

### *P. aeruginosa* forms non-attached aggregates in the apical layer of the epithelial cells

**Fig 2:**
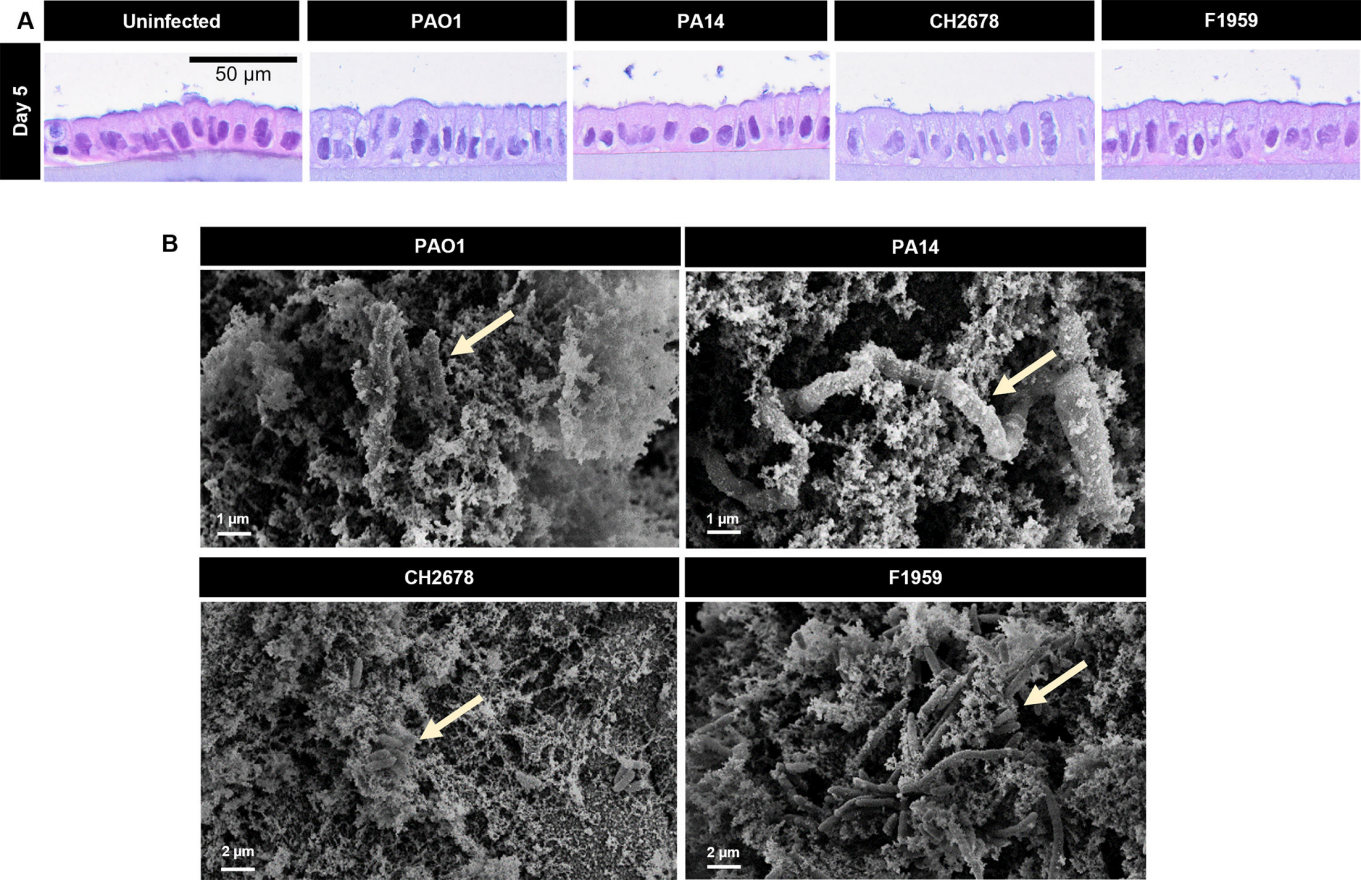
*P. aeruginosa* forms non-attached aggregates in the apical layer of the epithelial cells (**A**) Hematoxylin and eosin staining of paraffin-embedded Calu-3 cells infected with the four indicated *P. aeruginosa* strains at day 5 p.i. (scale: 50 µm). (**B**) (SEM images following partial preservation of mucus and other cellular materials by lysine acetate, ruthenium red, and osmium (LRR) fixation showing *P. aeruginosa* (arrows) forming non-attached aggregates within the apical layer above the epithelial cells at day 5 p.i. (scale: 1–2 µm). Data in this section were obtained from Calu-3 cells, which were infected with *P. aeruginosa* strains and protected with adapted concentrations of tobramycin in the basolateral compartment.

Histopathological imaging of infected Calu-3 cells showed maintained polarity, adhesion to the membrane, and structural integrity despite being infected with *P. aeruginosa* for 5 days (Fig. 2A). This was corroborated by scanning electron microscopy (SEM) images, revealing an intact differentiated epithelial layer with apical protrusions even after prolonged bacterial infection (Fig. S3). However, bacteria could not be visualized on the host cells. Since Calu-3 cells are known to produce mucus at ALI (41–43), we speculated that this might be due to loss of mucus and other cellular material at the apical side of the cells during sample preparation for both histopathological and SEM imaging. To address this, we applied a fixation protocol that has previously been developed to partially preserve the apical mucus (44–46) (Fig. S3). Using this protocol, we were able to locate the bacteria and confirm their presence as non-attached aggregates within the apical layer of the cells (Fig. 2B).

### *P. aeruginosa* upregulates expression of virulence factors upon extended contact with the airway epithelium

To elucidate the specific crosstalk between *P. aeruginosa* and the infected Calu-3 cells, we applied a dual-sequencing approach. Calu-3 cells were infected with PAO1, protected by the basolateral addition of tobramycin, and allowed to interact with the bacteria for 1 and 5 days prior to bacterial and eukaryotic RNA extraction and analysis. RNA was extracted in bulk from the infected Calu-3 cells, and we examined changes in bacterial gene expression over time by comparing the PAO1 gene expression profile at day 5 p.i. versus day 1 p.i. We found 835 differentially expressed genes (DEG). Interestingly, we observed an upregulation of genes involved in pathogenesis, cell division, and the protein secretion apparatus. This increase was dominated by an enrichment of genes of the type III secretion system (T3SS) (*pscN, pscP, pcr3, pcr1, pscQ*, and *popN*), the type VI secretion system, and pyoverdine biosynthesis. (Table 1; Table S1). A similar observation was made in the PAO1-infected NHBE cells when comparing day 3 versus day 1 of infection. There were 98 differentially expressed genes, which included upregulated genes involved in type III, and type VI secretion as well as pyoverdine biosynthesis (Table 1; Table S1).

**TABLE 1:** Upregulated genes in PAO1 at day 5 p.i. versus day 1 p.i. in Calu-3 cells and at day 3 p.i. versus day 1 p.i. in NHBE cells^*a*^

			Calu-3 (Day 5 vs Day 1)	NHBE (Day 3 vs Day 1)
	Locus tag	Gene	Log_2_FC	Log_2_FC
Type III secretion systems	PA0044	*exoT*	3.1	1.5
	PA1700	*pcr2*	4.6	1.8
	PA1707	*pcrH*	4.2	2.0
	PA1709	*popD*	3.2	1.7
	PA2191	*exoY*	3.4	2.0
	PA3841	*exoS*	2.0	1.5
	PA3842	*spcS*	3.8	1.8
	PA1694	*pscQ*	5.4	
	PA1695	*pscP*	5.8	
	PA1696	*pscO*	2.3	
	PA1697	*pscN*	6.3	
	PA1698	*popN*	5.3	
	PA1699	*pcr1*	5.5	
	PA1701	*pcr3*	5.5	
	PA1702	*pcr4*	4.9	
	PA1706	*pcrV*	4.2	
	PA1710	*exsC*	3.2	
	PA1711	*exsE*	3.1	NA
	PA1715	*pscB*	4.0	
	PA1716	*pscC*	3.7	
	PA1717	*pscD*	3.6	
	PA1718	*pscE*	3.5	
	PA1719	*pscF*	3.5	
	PA1720	*pscG*	3.0	
	PA1721	*pscH*	3.4	
	PA1722	*pscI*	3.2	
	PA1723	*pscJ*	3.2	
	PA1724	*pscK*	3.1	
	PA1725	*pscL*	2.8	
Pyoverdine biosynthesis	PA2389	*pvdR*	1.5	1.8
	PA2390	*pvdT*	1.7	2.2
	PA2391	*opmQ*	1.7	2.2
	PA2393	NA	3.9	3.3
	PA2385	*pvdQ*	2.0	
	PA2386	*pvdA*	2.8	
	PA2392	*pvdP*	3.2	
	PA2394	*pvdN*	3.5	
	PA2395	*pvdO*	3.1	
	PA2396	*pvdF*	2.8	NA
	PA2397	*pvdE*	2.9	
	PA2399	*pvdD*	1.8	
	PA2424	*pvdL*	1.7	
	PA2425	*pvdG*	2.6	
	PA2403	*fpvG*	NA	1.4
	PA2413	*pvdH*		2.9
Type VI secretion system	PA0091	*vgrG1*	2.0	
	PA0092	*tsi6*	1.6	
	PA0093	*tse6*	1.9	
	PA1661	*hsiH2*	2.1	
	PA1663	*sfa2*	2.2	
	PA1666	*lip2*	2.5	NA
	PA1668	*dotU2*	1.9	
	PA1669	*icmF2*	1.6	
	PA2684	*tse5*	1.9	
	PA2685	*vgrG4*	1.6	
	PA3486	*vgrG4b*	1.7	
	PA1511	*vgrG2a*		1.6
	PA3294	*vgrG4a*		1.4
	PA5266	*vgrG6*		2.1
	PA1512	*hcpA*	NA	1.8
	PA1658	*hsiC2*		1.8
	PA1660	*hsiG2*		1.3
	PA1662	*clpV2*		1.5
	PA5267	*hcpB*		1.9
Extracellular polysaccharide biosynthesis	PA3064	*pelA*	2.2	
	PA3063	*pelB*	2.0	
	PA3062	*pelC*	2.5	NA
	PA3061	*pelD*	2.8	
	PA3060	*pelE*	2.7	
	PA3059	*pelF*	2.3	

^*a*^ Calu-3 and NHBE cells were infected with PAO1 and protected with adapted concentrations of tobramycin. DEG indicates differentialy expressed genes and NA indicates that genes were not differentially regulated.

### *P. aeruginosa* colonization of host airway microenvironment results in an *in vivo*-like bacterial transcriptional profile

We then explored how the *P. aeruginosa* transcriptional profile observed in our model compares with those previously recorded upon *in vitro* and *in vivo* host cell contact in airway-relevant contexts. Although the majority of *P. aeruginosa* airway infections occur outside the context of CF (47), the only currently available transcriptomic data of *P. aeruginosa* lung infections were obtained from CF patients. Our model is not disease specific and aims to recapitulate the general core conditions of the infection microenvironment while enabling time-resolved studies. Nevertheless, we compared the *P. aeruginosa* transcriptome in our model to that in CF patients to evaluate it in the context of currently existing data.

We initially applied a previously published analytical framework to calculate an accuracy score (AS_2_) (48), and determined the percentage similarity of gene expression in our ALI model compared to those previously reported in 24 CF sputum samples (25). Our results showed that the AS_2_ score increased from 77% in planktonically grown PAO1 in cell culture medium to 84% in the day 5 p.i. culture of our Calu-3 ALI model, while the AS_2_ score of PAO1 infecting NHBE cells was 84% at day 1 and 83% at day 3 (Fig. S4). Hence, utilizing the Calu-3 cell line resulted in comparable scores as those seen using primary cells particularly at the late time point.

To further understand the observed expression profile within a broader context, we then performed a multidimensional scaling (MDS) analysis of the PAO1-infected samples in this study as well as those from other *in vitro* and *in vivo* data sets (25, 49) (Fig. S4). The Calu-3 ALI model clustered with the NHBE ALI model for both the early and late time points of infections. The transcriptional profiles of PAO1 in both our cell types clustered with those previously recorded in another ALI airway epithelial cell model (25). Notably, the transcriptional profiles observed upon prolonged treatment in our model shifted toward those recorded in the human CF lung environment (25, 49) rather than toward those observed in an *in vitro* ALI model utilizing synthetic CF sputum medium.

**Fig 3:**
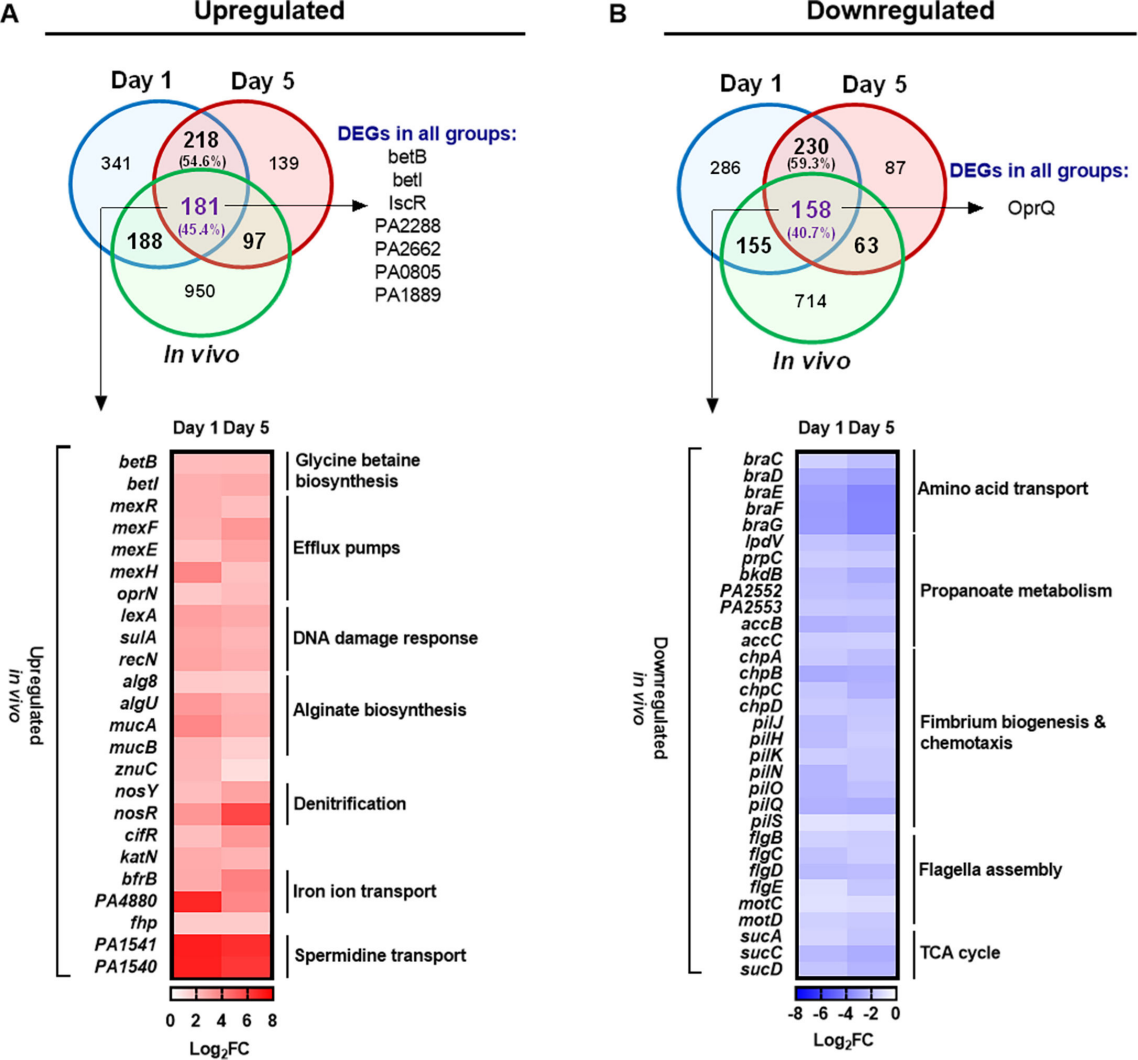
Commonly expressed *P. aeruginosa* genes in the ALI cultures of human epithelial cells and previously published CF patient *in vivo* transcriptomes. Number of upregulated (**A**) and downregulated (**B**) genes at day 1 p.i. and day 5 p.i. in Calu-3 ALI cultures as compared to planktonic growth (this study) that overlap with differentially regulated genes in at least one of three previously published *in vivo* transcriptomes (Kordes *et al.* [49], Rossi *et al.* [50], and Cornforth *et al.* [51]). The heat-map depicts gene expression values of a selection of the overlapping differentially expressed genes (full gene list can be found in Table S2). Genes that overlapped between both our time points and all three of the *in vivo* transcriptomes from CF patients are shown under the heading “DEGs in all groups.” Data in this section were obtained from Calu-3 cells, which were infected with PAO1 and protected with 100 µg/mL tobramycin.

We next aimed at identifying genes that were differentially regulated in our model and the CF lung environment. There are three studies in which the *in vivo* bacterial transcriptome in sputum and lung tissue of CF patients was compared to the *P. aeruginosa* transcriptional profile recorded in *in vitro* cultures grown under standard laboratory conditions (49–51). We generated a list of genes, which were differentially expressed during growth in our Calu-3 ALI model at day 1 and at day 5 p.i. in comparison to growth of PAO1 under planktonic culture conditions. Nine hundred twenty-eight genes were found to be upregulated and 625 downregulated on day 1, while 829 genes were upregulated and 538 downregulated at day 5. Among the commonly upregulated genes at both infection time points, we found 181 (45.4 %) that were also upregulated in at least one of the three *ex vivo* studies, and among the 388 commonly downregulated genes, 158 (40.7 %) were also downregulated in at least one of the *ex vivo* studies (Fig. 3A and B). Commonly upregulated genes were significantly enriched in spermidine transport, efflux pumps, alginate biosynthesis, DNA damage response, and glycine betaine biosynthesis (Fig. 3B). Meanwhile, among the commonly downregulated were genes involved in amino acid transport, propionate metabolism, TCA cycle, flagella assembly, fimbria biogenesis, and chemotaxis (Fig. 3B; Table S2). We also analyzed the genes that overlapped between both our time points and all three of the *in vivo* transcriptomes from CF patients. The seven genes upregulated in all groups included *betI* and *betB* from the glycine betaine biosynthetic process, while the outer membrane protein *OprQ* was the one common gene downregulated in all groups.

The same analysis was performed by generating a list of genes, which were differentially expressed during growth in our NHBE ALI model at day 1 and at day 3 p.i. in comparison to growth of PAO1 under planktonic culture conditions. The total number of DEGs was much lower than in the NHBE model compared to the Calu-3 model with 165 genes upregulated and 176 downregulated on day 1, and 104 genes upregulated and 47 downregulated at day 3 (Table S1). Among the commonly upregulated genes at both infection time points, we found 29 (45.3 %) that were also upregulated in at least one of the three *ex vivo* studies, and among the commonly downregulated genes, 13 (31.7 %) were also downregulated in at least one of the *ex vivo* studies (Fig. S5A ; Table S2). Commonly upregulated genes include those involved in efflux pumps (*mexX*), DNA damage response (*lexA*), and denitrification (*nosR, nosZ*). The outer membrane protein *OprQ* was again the one common gene downregulated in both time points of this cell type and the three *in vivo* studies (Fig. S5A; Table S2). This further confirms that the bacterial response in our Calu-3 ALI model is comparable to that observed when using more complex primary cell ALI cultures.

Hence, the transcriptional profile of *P. aeruginosa* infecting ALI cultures of human airway epithelial cells in our Calu-3 ALI model recapitulates the low energy, low motility, low growth, and high biofilm and activated stress responses observed in *P. aeruginosa* infections of the human CF lung.

### Prolonged *P. aeruginosa* infection results in a sustained host pro-inflammatory response

**Fig 4:**
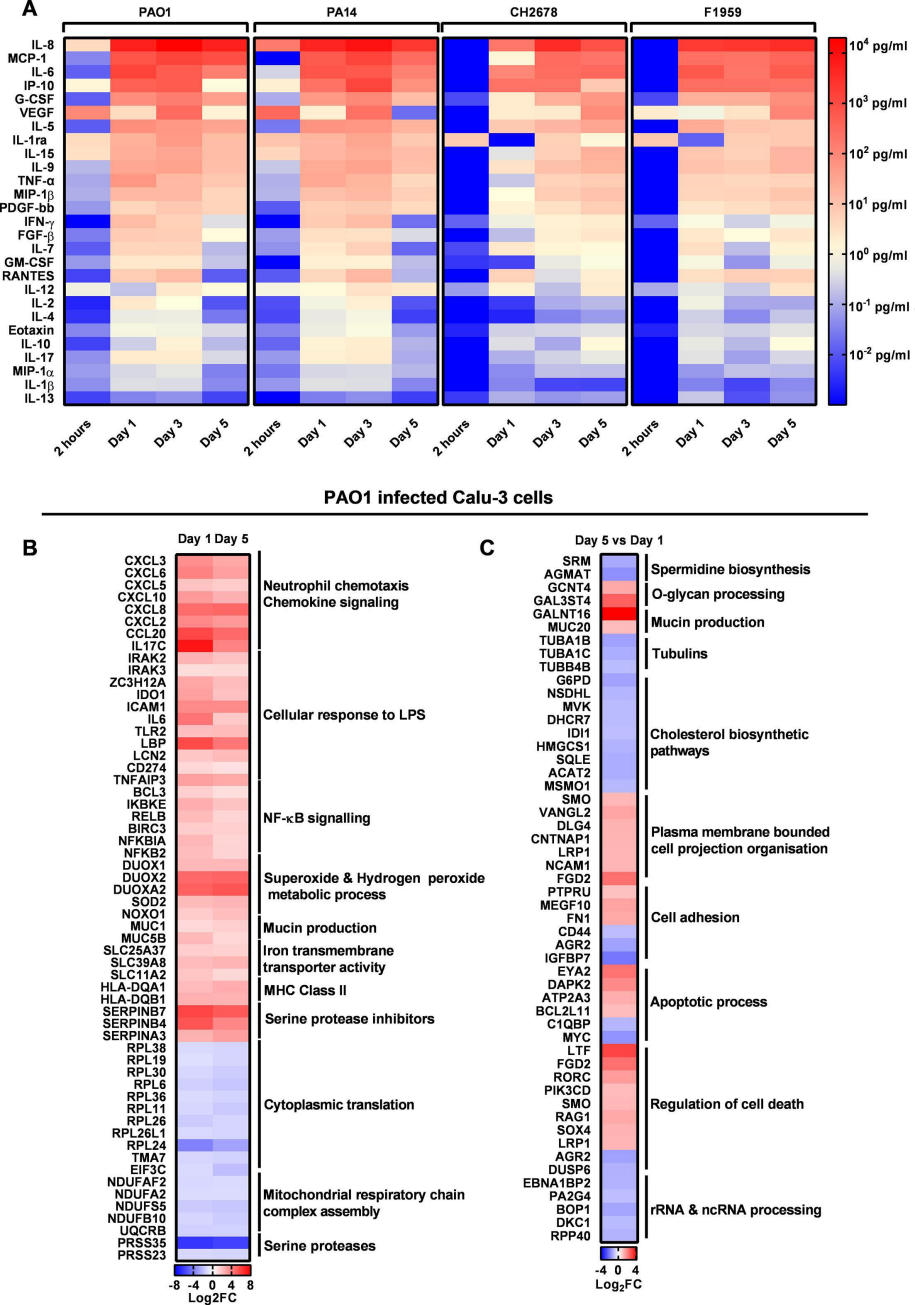
Prolonged *P. aeruginosa* infection results in a sustained pro-inflammatory response. (A) Screening of 27 cytokines produced by *P. aeruginosa*-infected Calu-3 cells as measured by the use of a magnetic bead-based immunoassay at four different time points and with four different *P. aeruginosa* strains. (B) A selected list of differentially expressed genes from day 1 and day 5 of PAO1-infected Calu-3 cells as compared to uninfected Calu-3 cells. (C) A selected list of differentially expressed genes at day 5 as compared to day 1 of PAO1-infected Calu-3 cells. Full gene lists can be found in Table S3. Data in this section were obtained from Calu-3 cells, which were infected with PAO1 and protected with 100 µg/mL tobramycin.

Infections of host Calu-3 cells, protected by adapted concentration of tobramycin at the basolateral side, with four strains of *P. aeruginosa* resulted in a sustained pro-inflammatory activation. Quantification of secreted cytokines in the cell culture medium showed a significant induction of IL-8, MCP-1, IL-6, IP-10, and G-CSF as measured by the use of a magnetic bead-based immunoassay up to day 5 p.i. (Fig. 4A). The clinical isolates CH2678 and F1959 induced a delayed and slightly attenuated cytokine response compared to PAO1 and PA14 (Fig. S1B). A similar pro-inflammatory phenotype was observed in PAO1-infected NHBE cells with IL-8, G-CSF, IP-10, IL-6, and TNF-α being the most highly expressed cytokines (Fig. S2C).

Analysis of the host cell RNA sequencing reads in our dual-seq approach also revealed significant changes in gene expression in Calu-3 cells on both day 1 and day 5 post PAO1 infection compared to corresponding uninfected cells as controls. Nine hundred seventy-four and 1,339 genes were differentially expressed on day 1 and day 5, respectively, in PAO1-infected Calu-3 cells (Table S3). Genes involved in neutrophil chemotaxis and chemokine signaling were upregulated with CXCL8, CCL20, and IL17C, showing the highest values. Genes involved in cellular response to lipopolysaccharide (LPS) were also consistently enriched, with IL-6, ICAM-1, and LBP showing the highest expression (Fig. 4B**;** Table S3). These changes in gene expression are in line with the observed persistent cytokine production over the course of infection.

Furthermore, dual oxidase genes (DUOX1, DUOX2, and DUOXA2) were upregulated in infected cells indicating the activation of hydrogen peroxide metabolic processes. Genes coding for serine protease inhibitors (SERPINB7 and SERPINB4) and mucin production (MUC1 and MUC5B) were also significantly enriched. Finally, several genes of the nuclear factor-κ B (NF-κB) signaling pathway (NFKBIA, NFKB2, BCL3, IKBKE, RELB, and BIRC3) were also upregulated (Fig. 4B; Table S3). DEGs unique to the group day 5 versus day 1 in PAO1-infected Calu-3 cells showed a downregulation in genes involved in spermidine biosynthesis and an enrichment in upregulated genes involved in O-glycan processing and mucin production (Fig. 4B; Table S3). A similar pro-inflammatory profile was observed in the PAO1-infected NHBE cells when compared to uninfected controls. Here, we also observe an upregulation in genes involved with neutrophil chemotaxis, NF-κB signaling pathway, and cellular response to lipopolysaccharides (Fig. S5B).

In conclusion, the cytokine screen revealed a consistent cellular response across various *P. aeruginosa* strains, despite differences in their elicitation dynamics. Additional mRNA sequencing confirmed a robust host cell response toward prolonged presence of *P. aeruginosa* that was maintained throughout the duration of the infection and was overall less dynamic as compared to the bacterial response.

## DISCUSSION

*P. aeruginosa* is a highly adaptable pathogen that owes much of its versatility to its large genome, facilitating precise regulation of gene expression in response to a variety of external cues (52, 53). To explore *P. aeruginosa* adaptation to challenging conditions during chronic respiratory tract infections, we established an ALI model of *P. aeruginosa* infection and employed a dual-seq approach to monitor both host and pathogen responses following prolonged host–pathogen interaction. Our findings reveal that upon infecting the airway epithelium, *P. aeruginosa* forms non-attached aggregates, adopts lower motility, and transitions toward a state of reduced energy consumption and growth, while enhancing pathogenicity traits. On the host side, the bacteria trigger a strong inflammatory response that is maintained throughout prolonged host-bacteria interaction. This transcriptional response is accompanied by a cytokine production profile that remains consistent across infections with various *P. aeruginosa* strains, although we observed differences in the dynamics of eliciting the response.

Bacterial persistence in chronic infections is often linked to the development of bacterial biofilms, characterized by bacteria adhering to surfaces and encapsulated within a thick self-produced extracellular matrix (54, 55). However, *in vivo* biofilms differ from those seen on non-living surfaces. Specifically, within the human airways, *P. aeruginosa* tends to form smaller, less complex aggregates surrounded by host material (49, 56). Likewise, in our model, bacterial interaction with the intact airway epithelium resulted in the formation of non-adherent bacterial aggregates within the apical layer of the cell. In accordance with a biofilm mode of growth, genes involved in bacterial motility were downregulated, while alginate biosynthesis genes were upregulated. Furthermore, prolonged contact between the host and bacteria resulted in an upregulation of the T3SS and the type VI secretion system (T6SS), similar to observations in the chronically infected CF lung (49). T3SS plays a major role in inducing host cell death, manipulating host immune responses, and disrupting the host cytoskeleton (57–60). Similarly, T6SS has been implicated in the internalization of *P. aeruginosa* into epithelial cells (61). Both T3SS and T6SS collectively were demonstrated to modulate virulence in type II pneumocytes (62), the *Galleria mellonella in vivo* infection model (63), murine models of pneumonia (6), and organoids infected at ALI (64). Moreover, we observed an increase in pyoverdine synthesis upon prolonged host–pathogen contact. Pyoverdine is a siderophore mediating iron acquisition as well as a signaling molecule (65, 66), which was demonstrated to be a crucial factor in *P. aeruginosa* virulence in various hosts (67, 68), and found to be highly expressed in the respiratory tract of CF patients (49).

Competition for iron is a well-described feature at host–microbe interfaces, both early and late in infections (6, 69–71). Accordingly, on the host side, we observed an increase in genes encoding iron transmembrane transporters, and on the bacterial side, an increase not only of siderophore biosynthesis but also an enrichment of genes involved in the acquisition and transport of heme over the course of infection. A direct reciprocal influence on gene expression could also be observed for the biosynthesis of spermidine. Spermidine is an endogenous host signal that influences infection outcomes *in vitro* (38, 72). Interestingly, the most upregulated genes in the bacteria were those involved in spermidine transport, while in the host, a downregulation of spermidine biosynthesis was observed.

Although our ALI model does not contain immune cells, epithelial cells are part of the first innate immune defense and are able to mount pro-inflammatory responses (73). NF-κB, a family of transcription factors required for the transcription of key proinflammatory processes in most cells, was consistently upregulated in our infection model (74). This mirrors previous findings where *P. aeruginosa* has been shown to activate NF-κB pathways in epithelial cells leading to the production of proinflammatory cytokines, enhanced bacterial clearance, mucin production, and neutrophil recruitment (75–78). The observed increased expression of genes involved in neutrophil chemotaxis is in line with the highly neutrophilic environment seen *in vivo* (79). Additionally, upregulation of the dual oxidase DUOX genes in this model reflects previous findings showing that *P. aeruginosa* activates DUOX resulting in hydrogen peroxide production in respiratory epithelial cells (80). We also see an elevated expression of mucins, both the secreted gel-forming MUC5B and the membrane-bound MUC1, indicating a cellular response geared toward increasing barrier protection. The infected epithelial cells showed high cytokine expression dominated by IL-8 and IL-6 in all tested strains with the CF isolates showing a delayed response in the earlier time points. This expression was robustly sustained until the last time point, indicating that when cell death is controlled, epithelial cells are capable of a continued and consistent cytokine production lead by IL-8 and IL-6 regardless of the infecting strain. High expression of IL-8 and IL-6 has been observed in chronically infected patients and in *in vitro* studies using strains isolated from later-stage infections, suggesting the cytokine profile observed here is in line with that observed in chronic infections (81–83). Overall, the epithelial cells showed infection-relevant inflammatory and protective responses to *P. aeruginosa* infections that were successfully sustained for several days.

In summary, our study provides a comprehensive description of the bidirectional impacts of persistent airway colonization. Moreover, we introduce an accessible infection model conducive to multifaceted modulation, enabling targeted exploration of the determinants underlying host-bacteria interactions. The established protocol is amenable to modulations to increase complexity, for example, by adding immune cells (18, 84), commensal isolates (84), or other antibiotics (8). Evaluating these targets within *in vitro* models that accurately replicate infection-related conditions holds great promise for unraveling the intricacies of host–pathogen dynamics that may be a subject of change in different patients, each of whom harbors diverse microbiota that could potentially modulate the disease (85, 86) as well as distinct *P. aeruginosa* strains.

## MATERIALS AND METHODS

### Calu-3 cell culture

Uninfected Calu-3 cells (ATCC HTB55) were cultured in Dulbecco’s Modified Eagle Medium (DMEM; Gibco 41965039) supplemented with 10% heat-inactivated fetal bovine serum (FBS) (Sigma F7524). All cultures were maintained at 37°C and 5% CO_2_. ALI cultures were established on Nunc polycarbonate cell culture inserts for 24-well plates with a surface area of 0.47 cm^2^ and a pore size of 0.4 µm (Thermo Scientific 141002). The cell insert was set in the placeholder at the medium height notch, and 1 mL of cell culture medium was added to the basolateral compartment. Five hundred microliters of Calu-3 cell suspension was added to the apical compartment at a density of 4 × 10^4^ cells/cm^2^. The cells were incubated for 48 hours, after which the medium in the apical compartment was removed to set the cultures to the ALI. The basolateral medium was refreshed every 48–72 hours, and any medium observed in the apical compartment was removed. The cultures were maintained at the ALI for an additional 8–10 days, after which they were ready to be infected. All cultures were maintained at 37°C and 5% CO_2_.

### Calu-3 cell infection

Calu-3 cell infections were conducted using cell culture medium made from DMEM high glucose, no glutamine, no phenol red (Gibco 31053028) supplemented with 10% heat-inactivated FBS and 2 mM L-glutamine (Gibco 25030081). Four strains of *Pseudomonas aeruginosa* were used, namely PAO1, PA14, CH2678, and F1959. Overnight cultures were prepared by inoculating three colonies of each strain from sheep agar plates into 3 mL of lysogeny broth (LB) and incubating it in a shaking incubator at 37°C, 180 rpm, for 18 hours (or overnight). Overnight cultures were then diluted 1:100 in 3 mL cell culture medium (described above) and incubated at 37°C, 180 rpm, for another 3–4 hours or until the cultures reach an OD_600_ of around 0.1–0.3. One milliliter of this culture was centrifuged at 10,000 × *g* for 3 min, the supernatant was removed, and the bacterial pellet was re-suspended in 1 mL of fresh cell culture medium. This bacterial suspension was diluted 1:10 in cell culture medium and OD_600_ was measured. The bacterial suspension was further diluted to make an inoculum solution with a density of 1 × 10^5^ bacteria/mL.

Two hundred microliters of the bacterial inoculum solution was added to each insert containing differentiated Calu-3 cells (2 × 10^4^ bacteria/well, or 4.3 × 10^4^ bacteria/cm^2^), centrifuged at 200 × *g* for 5 min, and incubated for 2 hours. Afterward, the inoculum was removed to reinstate ALI conditions, and basolateral medium was substituted with 1 mL of cell culture medium containing tobramycin (Sigma-Aldrich T1783). The infection was maintained for 5 days with tobramycin-containing media which was refreshed at day 1 and day 3. The optimized concentration of tobramycin used was 100 µg/mL for PAO1 and 130 µg/mL for PA14 and the CF isolates CH2678 and F1959. All cultures were maintained at 37°C and 5% CO_2_.

### NHBE cell culture

NHBE cells (CC-2540S, Lonza; Walkersville, MD) were cultured according to the manufacturer’s instructions. Briefly, donor cells were thawed and seeded into 75 cm^2^ cell culture flasks at a density of 5 × 10^5^ cells per flask. After a 1-week expansion phase, cells were seeded onto 24-well rat tail collagen-coated Transwell membranes (Corning) with a pore size of 0.4 µm at a density of 3.3 × 10^4^ cells per well. After submerged cultivation for 1 week in PneumaCult EX medium (Lonza) (supplemented with PneumaCult EX 50× supplement, hydrocortisone [96 ng/mL], and fungin [20 µg/mL]), membranes were air-lifted and the culture medium in the basolateral compartment changed to PneumaCult ALI (Lonza) (supplemented with PneumaCult ALI 10× supplement, ALI maintenance supplement, heparin[(4 µg/mL], hydrocortisone [480 ng/mL], and fungin [20 µg/mL]). Medium in the basolateral compartment was refreshed every 48–72 hours, and mucus was removed once per week by gentle washing with phosphate buffered saline (PBS) and aspiration. Differentiation into a pseudostratified epithelium was complete after 5 weeks under ALI conditions and was evaluated microscopically by occurrence of cilial beating and mucus production. All cultures were maintained at 37°C and 5% CO_2_.

### NHBE cell infection

One hundred microliters of a glycerol stock of PAO1 (DSMZ #19880) was inoculated into 50 mL LB medium and incubated for 16 hours at 150 rpm on a shaking incubator at 37°C. These LB overnight cultures were centrifuged at 3,000 × *g* for 10 min and the pellets resuspended in 10 mL PBS. After dilution to an OD_600_ of 0.02 (equal to 2 × 10^6^ colony-forming units [CFU]/mL), 50 µL of the bacterial suspension or PBS for the uninfected control was added to the apical side of NHBE membranes. Cells were centrifuged at 300 × *g* for 5 min (37°C) to enhance contact between host cells and bacteria. After 1 hour incubation at 37°C and 5% CO_2_, inoculum was removed and the cells post-incubated to the desired time points. Ten to 20 µg/mL tobramycin was added to the basolateral medium 6 hours p.i. All cultures were maintained at 37°C and 5% CO_2_.

### Determination of cell viability

Calcein AM fluorescent dye (Corning 354217) was reconstituted according to the manufacturer’s instructions and diluted to a working concentration of 80 µM in the cell culture medium (Calu-3 cells) or PBS (NHBE cells). Calcein AM solution was added apically (500 µL for Calu-3 and 100 µL for NHBE) and incubated at 37°C, in a shaking incubator (180 rpm for Calu-3 and 150 rpm for NHBE) for 45 min in the dark. Subsequently, the staining solution was removed, and lysis solution of 1% Triton-X was added to the stained cells (250 µL for Calu-3 and 200 µL for NHBE). The plate was incubated for an additional 30 min at room temperature in the dark. Cell lysates were further homogenized by pipetting, and duplicates of 50 µL/well of the solution were added to a black 96-well plate. Fluorescence was measured at 494/517 nm (Abs/Em) in a plate reader (BioTek Synergy H1). Percentage viability was determined by the following calculation: [(infected/uninfected) × 100].

### Bacterial detection and quantification

Bacterial presence was determined by spotting apical secretion or basolateral medium on LB agar. Apical bacterial load was quantified by lysing the infected Calu-3 or NHBE cells as described above, serially diluting the lysate in PBS, and plating on LB agar. LB agar plates were incubated at 37°C for 18 hours. The number of colonies were counted, and CFU were calculated.

### Determination of apical tobramycin concentrations (spot assay)

Bacterial lawns were prepared for PAO1 and PA14 by diluting three colonies of bacteria grown on sheep blood agar into 3 mL of PBS, spreading 1 mL of this solution onto LB agar plates, and allowing them to dry. Then, 3 µL of tobramycin-containing cell culture media at increasing concentrations was spotted onto the bacterial lawn and allowed to dry. The spotted plates were incubated at 37°C for 16 hours, and the diameter of the inhibition zones was measured. A standard curve was determined for each strain, indicating a linear relationship between tobramycin concentration and the diameter of the inhibition zone up to a concentration of 130 µg/mL. The linear equations were determined to be y = 0.0589x for PAO1 and y = 0.0566x for PA14. Subsequently, the above-mentioned Calu-3 culture and infection protocol was applied with an empty inoculum (no bacteria) at the optimized tobramycin concentrations of 100 µg/mL for PAO1 and 130 µg/mL for PA14. Three microliters of the apical liquid from each condition was spotted onto a lawn of either PAO1 or PA14, respectively, and incubated at 37°C for 16 hours. The diameter of the zone of inhibition was measured and the linear equations were used to determine the approximate apical tobramycin concentration. Data were generated using three to five biological replicates per condition.

### Hematoxylin and eosin (H&E) staining

H&E staining was performed on Calu-3 cells infected with the above-mentioned four strains of *P. aeruginosa* treated with the respective optimized tobramycin concentrations at the basolateral compartment (see above). Membranes with ALI-cultured Calu-3 cells were fixed in 4% buffered formalin and embedded in paraffin according to standard histopathological protocols. Sections of 4 µm–5 µm thickness were taken from the paraffin-embedded samples and stained with hematoxylin and eosin. Sections were evaluated on a light microscope (BX43, Olympus) equipped with 4×–40× objectives and a digital camera (SC50, Olympus).

### Scanning electron microscopy

Electron microscopy (EM) was performed on Calu-3 cells infected with the above-mentioned four strains of *P. aeruginosa* treated with the respective optimized tobramycin concentrations (see above). Sample preparation was performed as previously described with two different fixation protocols (87). In brief, cells on culture inserts were either fixed with (i) EM HEPES buffer with 5% formaldehyde and 2% glutaraldehyde for 1 hour (standard protocol), or (ii) in a stepwise process starting with 0.2 M cacodylate pH 6.9 plus 0.15% ruthenium red, 2% formaldehyde, 2.5% glutaraldehyde, and 75 mM l-lysine acetate (30 min on ice), followed by two washing steps with 0.2 M cacodylate pH 6.9 with 0.15% ruthenium red and a second fixation without l-lysine acetate for 2 hours on ice (LRR protocol) (88). Afterward, the inserts were washed two times and treated with OsO_4_ (800 µL washing solution + 200 µL 5% OsO_4_ aq.). To avoid membrane damage, dehydration was carried out with ethanol instead of acetone. Samples were processed in a gradient series on ice (10%, 30%, 50%, 70%, and 90%), each step for 10 min followed by two steps at room temperature with 100% EtOH. Critical point drying and sputter coating with gold palladium were performed with a CPD300 from Leica and a SCD 500 from Bal-Tec. A field-emission scanning electron microscope (Merlin from Zeiss) at an acceleration voltage of 5 kV and an Everhart Thornley HESE2/inlens SE detector ratio of 25:75 or 50:50 was used.

### Cytokine measurement

Cytokine concentrations were determined using Bio-Plex Pro Human Cytokine 27-plex Assay (Bio-Rad) following manufacturer’s instructions using the basolateral medium of Calu-3 and NHBE infected with the above-mentioned four strains of *P. aeruginosa* treated with the respective optimized tobramycin concentrations (see above). Fifty microliters of medium diluted 1:1 with sample diluent was used for the assays. Standards were reconstituted and prepared as described in the manufacturer’s instructions. Standard curves and concentrations were calculated using the Bio-Plex Manager 6.1 software. Data were obtained from three to five biological replicates per condition.

### RNA extraction, sequencing, and transcriptomic analysis

RNA was extracted from 1 mL of the PAO1 planktonic cultures from which the Calu-3 infection inoculum was prepared (from LB overnight cultures diluted 1:100 in cell culture medium and grown for 3 hours until OD_600_ of 0.1–0.3 as mentioned above), uninfected Calu-3 cells, PAO1-infected Calu-3 cells, as well as PAO1-infected NHBE cells at two different time points (three inserts per experiment). All experiments were done in triplicate, with the exception of the Calu-3 uninfected control at day 1, which was a duplicate. The Calu-3 and NHBE cells were infected and treated with tobramycin as described above. The RNeasy Mini Kit (Qiagen) in combination with Qiashredder columns (Qiagen) was used according to the manufacturer’s instructions. DNA was removed using the DNA-free Kit (ThermoFisher). Obtained RNA was quality checked using the RNA Nano Kit (Agilent Technologies) on an Agilent Bioanalyzer 2100 (Agilent Technologies). The removal of bacterial ribosomal RNA was performed using the Ribo-off rRNA Depletion Kit for Bacteria (Vazyme Biotech). Eukaryotic ribosomal RNA was removed using Ribo-off rRNA Depletion Kit (Human/Mouse/Rat) (Vazyme Biotech). cDNA libraries were generated with NEBNext Ultra II Directional RNA library prep kit (New England Biolabs) and sequenced on NovaSeq 6000 device. Bacterial sequencing reads were quality controlled, adapter clipped using cutadapt (89), and mapped to the reference genome of PAO1 (NC_002516.2) with bowtie 2 (90). Read counts of the genes were extracted with featureCounts (91) and used as the basis for further analyses. Sequencing reads of the host were processed and mapped to the human genome (GRCh38.p13) using the STAR aligner (92). Differential gene expression analysis was performed with the R package edgeR (v.3.20.1) (93). Normalization factors to scale the raw library sizes were calculated using counts per million (cpm) and the weighted trimmed mean of M-values (TMM) method (94). Differential gene expression was calculated using the edgeR function glmTreat, and *P*-values were corrected for multiple testing using the method by Benjamini and Hochberg (false discovery rate, FDR) (95). Only genes with an FDR ≤0.05 were considered differentially expressed. Differentially regulated gene expression profiles were determined by comparing the expression profile of *P. aeruginosa*-infected Calu-3 cells at day 5 p.i. to that of day 1 p.i. and NHBE *P. aeruginosa* infected NHBE at day 3 p.i. and day 1 p.i compared to uninfected controls . We furthermore compared the *P. aeruginosa* gene expression profile at day 1 p.i. and day 5 p.i. in the Calu-3 model and day 1 p.i and day 3 p.i. of the NHBE model to the gene expression profile of *P. aeruginosa* planktonic culture in DMEM cell culture medium.

### Comparison of bacterial transcriptional profiles

The lists of upregulated and downregulated genes from day 1 p.i. and day 5 p.i. of our PAO1-infected Calu-3 model as well as day 1 p.i. and day 3 p.i. of our PAO1-infected NHBE model versus planktonically grown PAO1 in DMEM were then compared to the differentially regulated genes as published in three previous studies that compared the *P. aeruginosa in vivo* transcriptomes from CF sputum (50–51) and an explanted CF lung (49) to that of planktonically grown *P. aeruginosa*. DEGs from Cornforth et al. (51) were obtained from the comparison of CF sputum vs *in vitro*, where *in vitro* cultures were grown in chemically defined media at 37°C with shaking at 225 rpm. Common DEGs shared between cluster I (lungs) vs cluster II (lab. exp.) and cluster I (lungs) vs cluster III (lab. stat.) were used from Rossi et al. (50), which grouped DEGs in the lungs vs both stationary and exponentially growth phases in the corresponding *in vitro* cultures of *P. aeruginosa* grown in LB medium at 37°C with shaking at 150 rpm per minute. DEGs from the group *ex vivo* pool vs *in vitro* pool were used from Kordes et al. (49) where *in vitro* cultures were cultivated in LB media (inoculation with OD_600_ = 0.05) under shaking conditions (180 rpm) to early stationary phase (OD_600_ = 2). DEGs were set at log 2 fold chain (Log_2_FC), Log_2_FC ≥ 1.3 or Log_2_FC ≤ −1.3, and adjusted *P*-value <0.05.

Clustering analysis was performed using gene reads from this study as well as from Kordes et al. (49) and Lewin et al. (25). Data were normalized using the R package edgeR, and calculated using cpm and the weighted TMM method (94). Core genes were selected based on the annotation table available on pseudomonas.com (96), and genes should have at least 100 normalized reads in any sample. The MDS plot was generated using the R function plotMDS from the edgeR package and it was visualized using ggplot2.

AS_2_ analysis was performed as described in Lewin et al. (48) using the R package DESeq2 for variance stabilizing transformation normalization. Core genes were selected based on the annotation table available on pseudomonas.com (96) and selected genes should have at least 100 normalized reads in any strain. The script available at https://github.com/glew8/PA_ModelAccuracy/blob/main/AS2.R was used utilizing the function “score_target_vs_model_PA.” The resulting penalty between *in vivo* and *in vitro* samples was used to calculate the AS score, generating a percentage of genes with a penalty < |2| and a mean percentage of all samples in a group.

### MIC measurement

Overnight cultures were prepared by inoculating three colonies of each strain from sheep agar plates into 3 mL of LB and incubating it in a shaking incubator at 37°C, 180 rpm, for 18 hours (or overnight). Overnight cultures were then diluted 1:100 in 3 mL LB and incubated at 37°C, 180 rpm for another 3–4 hours. This day culture was diluted and mixed with tobramycin containing LB to make a final OD_600_ of 0.005 and incubated at 37°C, 180 rpm, for 18 hours. MIC was defined as the concentration of tobramycin with visible no bacterial growth.

## Data Availability

RNA-seq data from this study are available in the NCBI Gene Expression Omnibus (GEO) data repository under accession number GSE262433.

## SUPPLEMENTAL MATERIAL

Fig. S1 iai.00603-24-s0001.tif A: MICs of the *P. aeruginosa* lab strains PAO1 and PA14 and clinical isolates CH2678 and F1959 to the antibiotic tobramycin. B: Z-score of total cytokine production grouped by strain and time in Calu-3 cells infected with the 4 strains of *P. aeruginosa* as per the optimized protocol.

Fig. S2 iai.00603-24-s0002.tif Additional data on PAO1 infected NHBE at ALI.

Fig. S4 iai.00603-24-s0003.tif MDS plot of *P. aeruginosa* gene expression in various samples.

Fig. S5 iai.00603-24-s0004.tif Additional transcriptomic data of PAO1 infected NHBE cells at ALI.

Supplemental material iai.00603-24-s0005.docx Supplemental figure legends.

Table S1 iai.00603-24-s0006.xlsx Differentially expressed genes in PAO1 from infected Calu-3 and NHBE cells.

Table S2 iai.00603-24-s0007.xlsx DEGs in from PAO1 in both the Calu-3 and NHBE model and how they overlap with 3 in vivo studies in human lung infections.

Fig. S3 iai.00603-24-s0008.jpeg Additional SEM images of *P. aeruginosa* infected Calu-3 cells at ALI.

Table S3 iai.00603-24-s0009.xlsx DEDs from Calu-3 cells infected with PAO1.

ASM does not own the copyrights to Supplemental Material that may be linked to, or accessed through, an article. The authors have granted ASM a non-exclusive, world-wide license to publish the Supplemental Material files. Please contact the corresponding author directly for reuse.
