# Supplementary figures and images for: Establishment and characterization of persistent Pseudomonas aeruginosa infections in air–liquid interface cultures of human airway epithelial cells
Source: Infect Immun. 2025 Feb 18;93(3):e00603-24. doi: 10.1128/iai.00603-24 (PMC11895474; doi:10.1128/iai.00603-24)

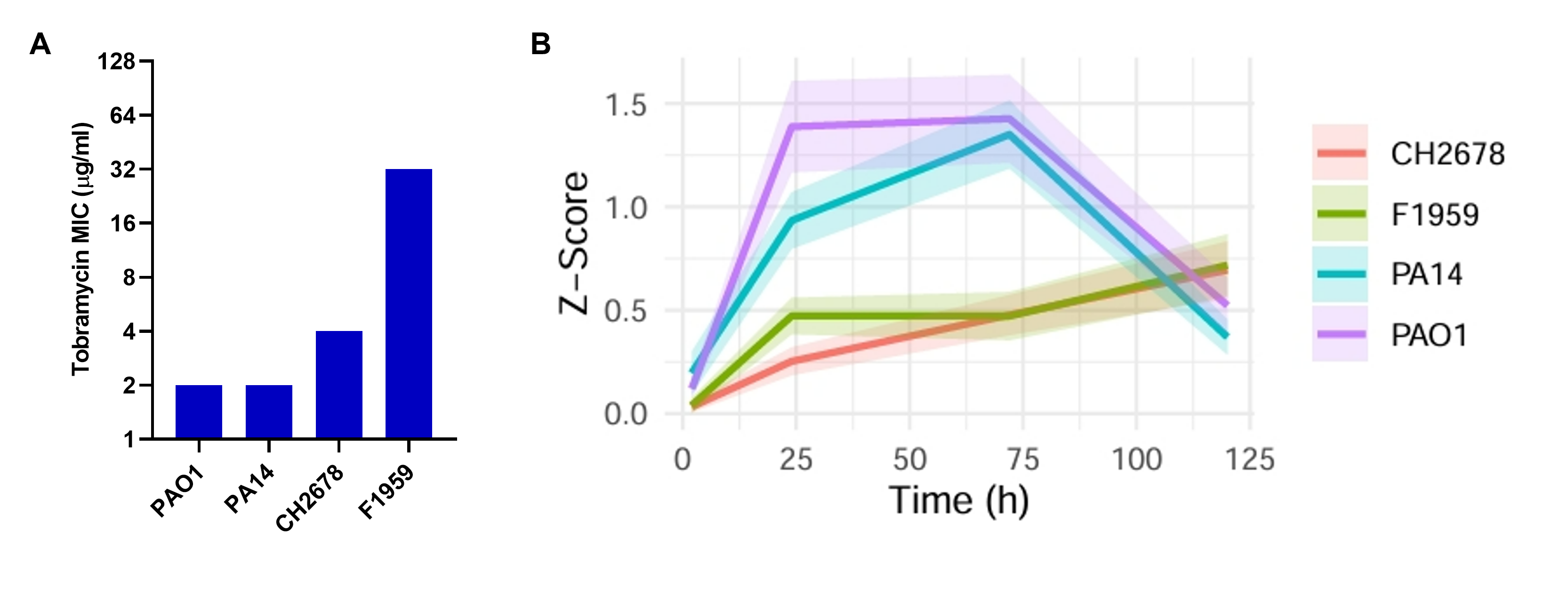

Supplement: Fig. S1 — A: MICs of the P. aeruginosa lab strains PAO1 and PA14 and clinical isolates CH2678 and F1959 to the antibiotic tobramycin. B: Z-score of total cytokine production grouped by strain and time in Calu-3 cells infected with the 4 strains of P. aeruginosa as per the optimized protocol. [file iai.00603-24-s0001.tif]

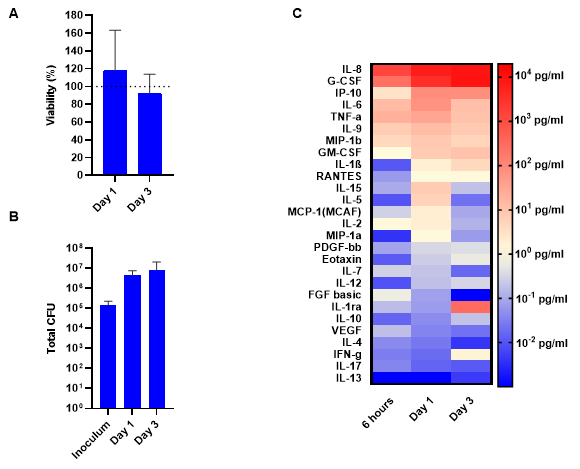

Supplement: Fig. S2 — Additional data on PAO1 infected NHBE at ALI. [file iai.00603-24-s0002.tif]

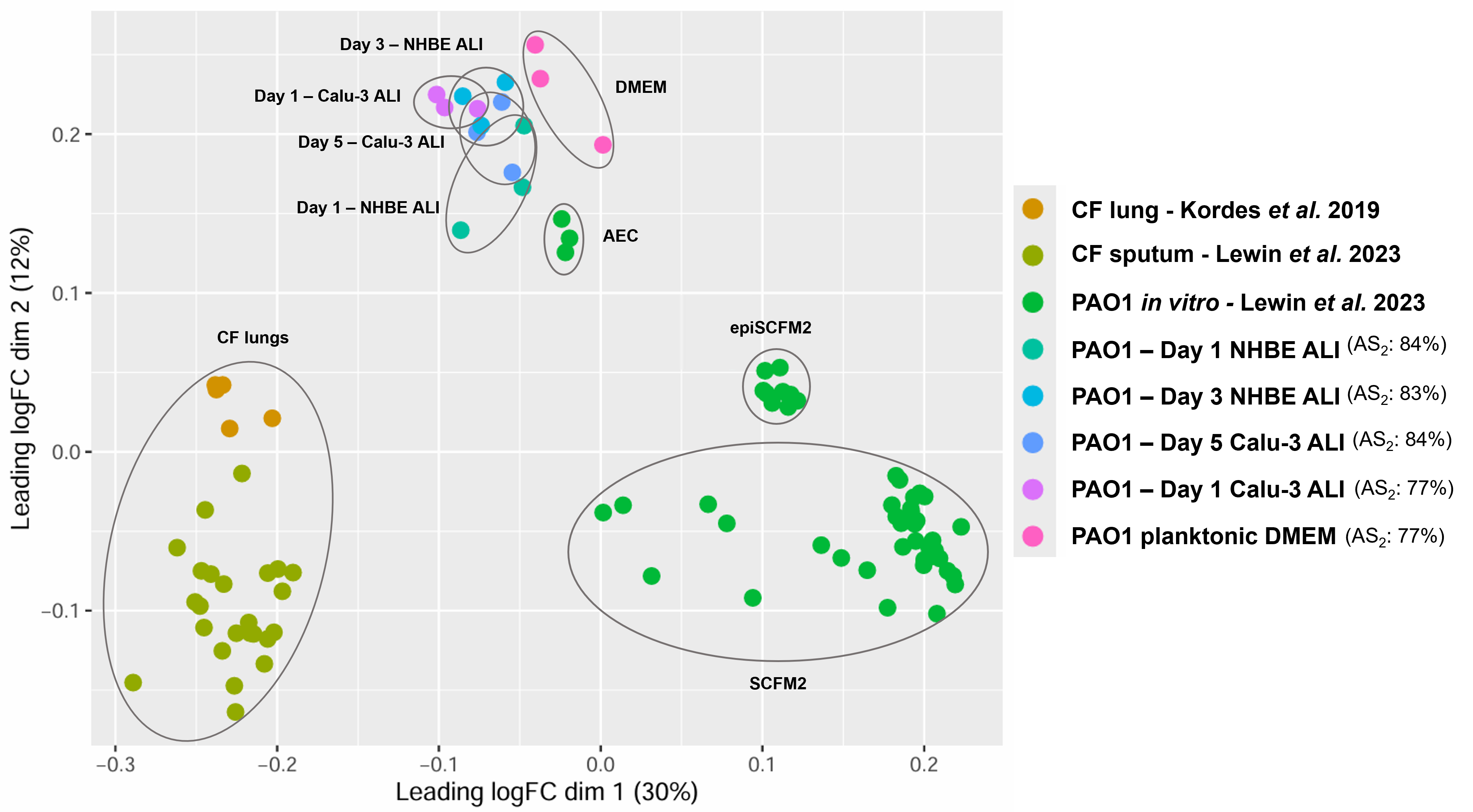

Supplement: Fig. S4 — MDS plot of P. aeruginosa gene expression in various samples. [file iai.00603-24-s0003.tif]

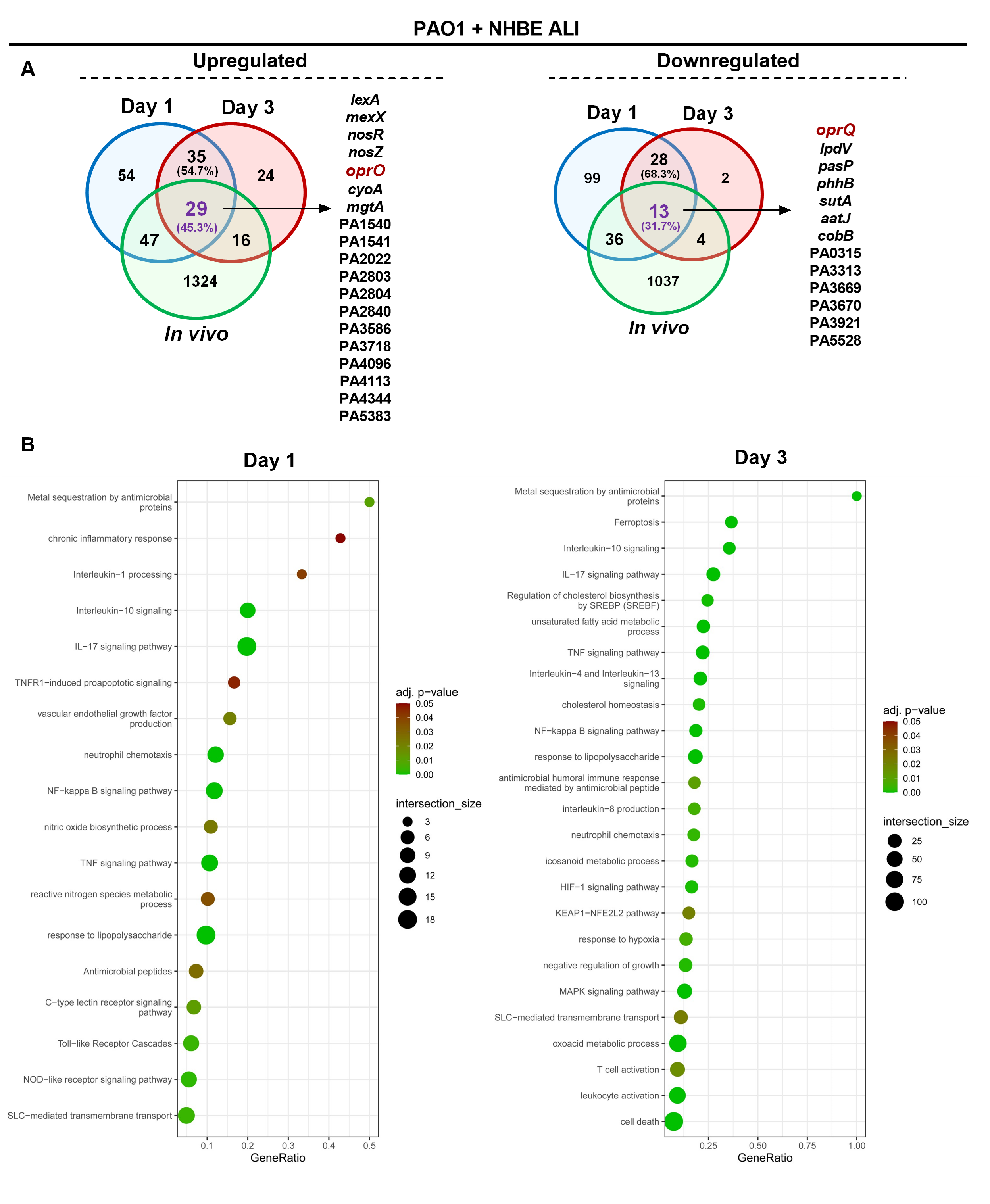

Supplement: Fig. S5 — Additional transcriptomic data of PAO1 infected NHBE cells at ALI. [file iai.00603-24-s0004.tif]

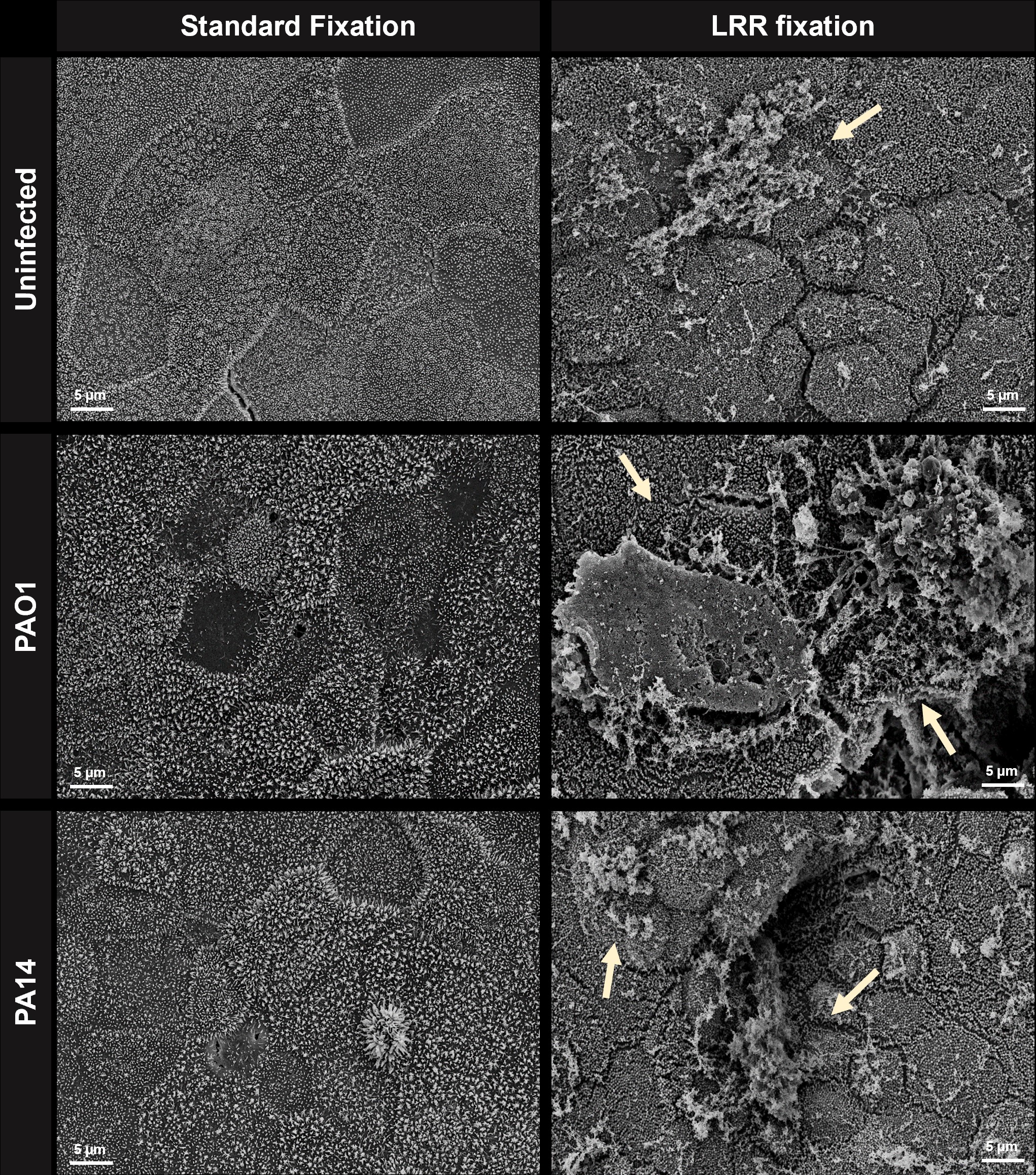

Supplement: Fig. S3 — Additional SEM images of P. aeruginosa infected Calu-3 cells at ALI. [file iai.00603-24-s0008.jpeg]
