# Supplementary material for: Establishment and characterization of persistent Pseudomonas aeruginosa infections in air–liquid interface cultures of human airway epithelial cells
Source: Infect Immun. 2025 Feb 18;93(3):e00603-24. doi: 10.1128/iai.00603-24 (PMC11895474; doi:10.1128/iai.00603-24)
Supplement: Supplemental material — Supplemental figure legends. [file iai.00603-24-s0005.docx]

**Supplementary Figure 1: A:** MICs of the *P. aeruginosa* lab strains PAO1 and PA14 and clinical isolates CH2678 and F1959 to the antibiotic tobramycin. **B:** Z-score of total cytokine production grouped by strain and time in Calu-3 cells infected with the 4 strains of *P. aeruginosa* as per the optimized ALI protocol. The solid lines indicate the mean and the shading around them is the 95% confidence interval. A lack of overlap between the colored shades around the lines indicates a significant difference with P- value <0.05.

**Supplementary Figure 2: *P. aeruginosa* infection of tobramycin treated NHBE cells.**

**A:** Viability of PAO1-infected NHBE cells treated basolaterally with 20 µg/ml tobramycin **B:** Total apical bacterial counts of PAO1-infected NHBE cells. **C:** Expression of 27 cytokines secreted by PAO1-infected NHBE cells. All data was obtained from 3 biological replicates per condition.

**Supplementary Figure 3: SEM images of Calu-3 cells fixed using the standard fixation (left) and LRR fixation protocol (right).** Both fixation methods show an intact differentiated epithelial layer with apical protrusion in both the uninfected control and in Calu-3 cells infected with PAO1 or PA14 for 5 days. The LRR fixation method was able to partially preserve apical cellular material (arrows) (Scale: 5µm).

**Supplementary Figure 4: *P. aeruginosa* gene expression varies depending on experimental conditions.**

Multidimensional scaling (MDS) plot of previously recorded gene expression profiles of clinical *P. aeruginosa* CF isolates in CF sputum (Lewin *et al*. 2023) and in CF lung tissue (Kordes *et al*. 2019), as well as the reference strain PAO1 in a synthetic CF medium model (SCFM2), a CF airway epithelial cell-SCFM2 model (epiSCFM2), and airway epithelial cells (AEC). Conditions from this study include PAO1 from day 1 and 5 of the infected Calu-3 ALI model, PAO1 from day 1 and 3 of the infected NHBE ALI model as well planktonically grown PAO1 in cell culture medium (DMEM). Accuracy score (AS_2_) defined as the percentage of genes expressed in conditions from this study compared to the expression profile in 24 CF sputum samples (Lewin *et al*. 2023) is indicated in the figure legend.

**Supplementary Figure 5: A:** Number of upregulated and downregulated genes at day 1 p.i. and day 3 p.i. in NHBE ALI cultures as compared to planktonic growth (this study) that overlap with differentially regulated genes in at least one of three previously published *in vivo* transcriptomes (Kordes *et al.* 2019, Rossi *et al.* 2018, and Cornforth *et al.* 2018). The overlapping genes are listed in the figure. Genes that overlapped between both our time points and all three of the *in vivo* transcriptomes from CF patients are shown in red (full gene list can be found in **Supplementary Table 2**). **B**: Upregulated gene categories in PAO1-infected NHBE cells at day 1 and day 3 compared to uninfected controls. All data was obtained from 3 biological replicates per condition. Data in this section was obtained from NHBE cells, which were infected with PAO1 and protected with 20 µg/ml tobramycin. All data was obtained from 3 biological replicates per condition.
